# Processing of fair and unfair offers in the ultimatum game under social observation

**DOI:** 10.1038/srep44062

**Published:** 2017-03-09

**Authors:** Jutta Peterburs, Rolf Voegler, Roman Liepelt, Anna Schulze, Saskia Wilhelm, Sebastian Ocklenburg, Thomas Straube

**Affiliations:** 1 Institute of Medical Psychology and Systems Neuroscience, University of Münster, Von-Esmarch-Str. 52, 48149 Münster, Germany; 2 Department of Psychology, University of Münster, Fliednerstr. 21, 48149 Münster, Germany; 3 Institute of Psychology, German Sport University Cologne, Am Sportpark Müngersdorf 6, 50933 Cologne, Germany; 4 Department of Biological Psychology, Institute of Cognitive Neuroscience, Ruhr-University Bochum, Universitätsstraße 150, 44801 Bochum, Germany

## Abstract

Social context influences social decisions and outcome processing, partially depending on inter-individual differences. The present study investigated social context-dependent modulation of behavior and feedback processing in the ultimatum game (UG) in relation to inter-individual differences in social anxiety. Thirty-two healthy adults completed the UG both under social observation and without observation. Offers were allegedly either randomly generated by the computer or drawn from a pool of offers from previous human players. Overall, fewer unfair than fair offers were accepted. Observation decreased acceptance rates for unfair offers. The feedback-locked feedback-related negativity (FRN) but not the P3 was modulated by observation and fairness, with stronger differential coding of unfair/fair under observation. This effect was strongly correlated with individual levels of social anxiety, with higher levels associated with stronger differential fairness coding in the FRN under observation. Behavioral findings support negative reciprocity in the UG, suggesting that (implicit) social norms overwrite explicit task instructions even in the absence of (alleged) social interaction. Observation enhances this effect. Fairness coding in the FRN was modulated by observation as a function of social anxiety, supporting the notion that altered sensitivity to equality in a social context may contribute to social avoidance in socially anxious individuals.

Human behavior is greatly influenced by the social context and underlying explicit and implicit social norms. But how exactly does the social context shape decisions and behavior? A large body of research has investigated social decision making as a pivotal aspect of human behavior. One of the most common experimental tasks in this line of research is the Ultimatum Game (UG)^1^. In this game, two players have to divide a given amount of money or points amongst themselves according to a simple protocol. One player, the proposer, offers a portion of the money or points to the other, the responder. If the offer is accepted by the responder, each player receives the amount he was proposed to get. Crucially, if the offer is rejected, none of the players receive any money or points. The UG thus introduces a conflict between economic self-interest, that is, maximization of the own payoff, and social motives such as reciprocity and inequity aversion.

Previous work has shown that acceptance rates are substantially lower for unequal than for equal offers, even though responders can only maximize their payoffs by accepting all offers (e.g., refs 2). This has often been ascribed to a natural tendency towards fairness^2^. Rejection of unfair offers, also referred to as negative reciprocity, may reflect prosociality, emphasizing the importance of (implicit) social norms and social motives, as the rejecting individual is sacrificing his/her own gain in order to punish unfair behavior^5^. However, previous work has indicated that both fair behavior and perceptions of fairness depend upon normative expectations about what is fair or appropriate in a given situation^6^. If a proposer was derived of his free choice, unfair offers were accepted to a greater extent^7^. Moreover, proposers tended to evade offering fair shares when their actions could not be detected by responders, indicating that proposers wanted to *appear* fair rather than actually *be* fair^3^,^8^,^9^.

The concept of negative reciprocity in the UG implies that another party is punished by the rejection, underlining the interactive character of the game. Notably, reduced acceptance rates for unfair as compared to fair offers have also been shown for allegedly computer-proposed offers, albeit to a lesser degree than for human-proposed ones^4^,^10^,^11^,^12^. Two possible explanations come to mind. First, this pattern may reflect a tendency of the subjects to vary their responses. In line with this, very few subjects stick to the same response even if that would (economically) be the best option^1^,^2^. Second, this pattern might reflect the notion that social motives are so strong they actually persist in the absence of actual social interaction. Indeed, the mere notion of interaction, e.g., assuming feedback was provided by another individual, was shown to be sufficient to increase hemodynamic and electrophysiological responses to feedback^13^,^14^,^15^,^16^. What is more, such (alleged) interaction is not even a prerequisite for deeming a situation social. The mere presence of an audience has been shown to impact behavior, facilitating performance on easy or well-trained task, and hindering performance on complex or unfamiliar tasks^17^. The notion of being observed and evaluated while performing has also been reported to be associated with increased electrophysiological responses to errors, likely due to increased error significance^18^. Surprisingly, how presence of an audience affects UG performance has not yet been systematically investigated. In previous studies that included an additional party in the UG, this party was an - often inactive - third player or a punishing party rather than a designated observer (e.g., refs 3,19,20). From a theoretical standpoint, two scenarios are conceivable: social observation could lead to a stronger focus on task instructions and maximization of own payoff, resulting in decreased rejection rates, or to stronger emphasis on implicit social norms (fairness), resulting in increased rejection of unfair offers. One of the aims of the present study was therefore to clarify the effect of social observation on behavioral performance of the UG.

Aside from allowing investigation of behavioral aspects of social decision making, the UG can also provide insight into the neural mechanisms underlying fairness processing. Several studies have used electroencephalography (EEG) and event-related potentials (ERPs) to investigate brain responses to fair and unfair offers in the UG. A medial frontal negativity occurring approximately 250 to 350 ms after offer presentation was more pronounced for unfair as compared to fair offers, an effect that was stronger in subjects with high fairness concerns^21^. This medial frontal negativity can also be interpreted as manifestation of feedback-related negativity (FRN), an ERP component typically assessed in response to performance feedback^22^,^23^,^24^. Several studies have corroborated fairness coding in the FRN time-locked to offer presentation in the UG (e.g., refs 4,25,26). Interestingly, the offer-locked FRN has also been shown to predict the decision to reject unfair offers^25^ and may be modulated by the relationship between proposer and observer^27^.

In addition to the FRN, the offer-locked P3, a later relative positivity in the ERP which has been linked to stimulus evaluation and decision processes^28^,^29^ and later, integrative stages of feedback processing that allow for coding of outcome ambivalence^30^, may also code for fairness^25^. Moreover, the P3 appears to be sensitive to social exclusion^31^.

Intriguingly, individual differences have been shown to affect behavioral and neural responses in the UG. Previous studies have reported altered acceptance rates of unfair human- or computer-proposed as well as altered fairness coding in FRN and P3 in individuals with high trait anxiety, with self-esteem and impulsivity but not depression as potential moderating factors^4^,^12^. State happiness has been associated with increased acceptance of unfair offers^26^. Moreover, high trait negative affect has been shown to modulate FRN and P3b, potentially reflecting increased rumination and decreased motivation^26^.

Previous electrophysiological UG studies have focused on offer-locked ERPs and thus neural responses to cues signaling *potential* outcomes, given that subjects had to accept the offers before obtaining any points. However, the FRN is typically measured time-locked to outcome presentation, thereby also coding for reward prediction errors (e.g. refs 32 and 33). While the outcome following a response is fully predictable in the classic UG, it is still conceivable that neural responses to both offer and outcome are modulated by factors such as fairness, proposer identity, and social context. Indeed, social observation in the UG may shift emphasis (and thus attention and salience) to the feedback phase of each trial, given that there is not very much to see for the observer during offer presentation because at that point the subject has not yet responded. Only with feedback onset does the observer get to evaluate the subject’s response, which should be obvious to the subject. Therefore the present study aimed to investigate the impact of fairness, proposer identity, and social observation on both offer- and feedback-locked neural responses.

The present study used an EEG-adaptation of the classic UG task in order to assess audience effects on brain responses and behavior as a function of fairness (fair versus unfair offers), offer source (human or computer), and inter-interindividual differences. With regard to the latter, audience effects should be particularly relevant to individuals with higher levels of social anxiety, given that fear of negative evaluation and rejection in social situations are key characteristics of social anxiety. Therefore, the relationship between social anxiety and altered UG performance and neural responses under social observation was also explored. Both offer- and feedback-locked neural responses (FRN, P3) were assessed. Acceptance rates were expected to be lower for unfair than for fair offers^2^,^3^,^4^, an effect that could be either enhanced or reduced under social observation, depending on whether audience effects would shift the focus on task instructions or implicit social norms. With regard to neural responses, it was hypothesized that FRN and P3 in the offer- and feedback-locked ERPs would be sensitive to fairness and offer source, with increased FRN amplitudes for unfair as compared to fair offers, and increased P3 to human as compared to computer offers^4^. Moreover, in view of altered coding of feedback valence in the FRN and P3 in the presence of an observer^34^, social observation was expected to augment fairness coding in FRN and P3. Last, it was hypothesized that higher levels of social anxiety would be associated with more pronounced effects of observation on behavior and ERPs.

## Methods

### Subjects

Thirty-four healthy adult volunteers (10 male, 24 female) participated in the present study. Subjects were recruited at the Institute of Medical Psychology and Systems Neuroscience at the University of Münster by public advertisement and received course credit and/or monetary reimbursement for participation. Exclusion criteria were current or past neurological or psychiatric disorders and history of head trauma or unconsciousness. Mean age was 22.6 years ±4.1 (range 18 to 35 years), and mean educational attainment was 12.8 years ±0.5 (range 12 to 14 years). Thirty subjects were right- and two were left-handed as determined by self-report. All subjects had normal or corrected-to-normal vision and were naïve to the study’s intent. Since previous research has indicated that ultimatum game performance and feedback processing is modulated by trait anxiety^4^,^12^, and because the present task specifically involved a manipulation of social context (see below), subjects also completed the German version of the Liebowitz Social Anxiety Scale (LSAS; German version)^35^ in order to determine individual levels of social anxiety. This questionnaire features 24 situations that are typically unpleasant for socially anxious individuals, and participants were asked to estimate their subjective levels of anxiety and avoidance for each situation on Likert scales ranging from zero (no anxiety/avoidance) to three (severe anxiety/avoidance). Avoidance and anxiety scores are added for a LSAS total score. Mean LSAS total score was 18.56 ± 12.05 (range 0 to 43). Total scores >60 are commonly found in clinical samples and are associated with pathological (generalized) social anxiety disorder^36^. Along these lines, the present sample presented with mild to no social anxiety. Moreover, subjects also completed the German version of the Beck Depression Inventory II (BDI-II)^37^ in order to assess possible existence and severity of depression symptoms. Mean BDI score was 2.70 ± 3.94 (range 0 to 11), thus yielding no evidence of depression in the present sample.

Written informed consent was obtained from all subjects prior to participation. The study conforms to the Declaration of Helsinki and has received ethical clearance by the Ethics Board of the German Society for Psychology (Deutsche Gesellschaft für Psychologie, DGPs).

As explained in more detail below, data from one subject had to be excluded from all analyses due to technical problems with response logging, rendering a sample on N = 33 for analysis of behavioral data. Furthermore, EEG data from one subject were excluded post-hoc due to off-label use of beta blockers, EEG data from two other subjects still contained artifacts after extensive preprocessing and were thus excluded, and EEG data from five other subjects had to be excluded because fewer than five trials were available for averaging in at least one condition. Overall, data from a sample of N = 26 subjects were entered into EEG analysis.

### Experimental task

The experimental task was a variant of the classic UG^1^ and adapted from previous electrophysiological studies^4^,^12^. Generally, in the UG, subjects are presented with offers on how to split an amount of money or points between two parties, that is, the subject as the responder, and another player as the proposer. Subjects have to decide whether to accept or reject the offers, but are informed that points or money will only be awarded to the parties in case of acceptance and not upon rejection.

In the present study, the social nature of the task was emphasized twofold: first, participants were informed that offers could be either randomly generated by the computer or drawn from a pool of offers that had been collected from previous participants. It was emphasized that it was thus impossible to identify individual proposers or to determine if multiple offers had been provided by the same individual, and that proposers would never know if their offers were rejected or accepted. This was done to eliminate retaliation as a potential confounding factor. Second, participants completed the task in two experimental conditions, once under social observation and once in a control condition without observation. In the observation condition, a non-transparent curtain that separated participant and experimenter was pulled aside and subjects were informed that an observer who was seated in direct view to the right of the participant would watch them very closely during the experiment and take notes. Staff members of the Institute of Medical Psychology and Systems Neuroscience served as observers and were standardized by wearing a white lab coat. Additionally, at the beginning of the test session, a webcam located on top of the subject’s screen was switched on and subjects were shown a live video feed of themselves. They were informed that the observer would be able to both directly see them and use the video feed to carefully observe their behavior and performance during the task. In the control condition, the curtain between experimenter and participant remained closed, and subjects were informed that the experimenter was merely present to ensure proper functioning of the technical equipment, and that he would not be monitoring their performance in any way.

Conditions were completed in two separate sessions which took place 7 to 14 days apart. Order of conditions was balanced across subjects. In order to ensure sufficient acceptance rates for all offer types and increased motivation, subjects were informed that their final payment after the second session would depend on their choices in the task. Specifically, they were told that, in addition to guaranteed course credit, they would receive monetary reward if their scores in each run exceeded 7000 points, with 7€ paid out for 7000 points and larger amounts of money for higher scores, respectively. The maximum score was 9000 points per condition.

**Figure 1:**
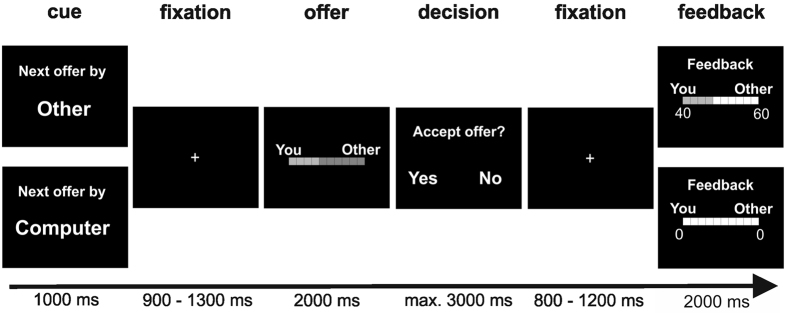
Schematic illustration of the time course of stimulus presentation in the present ultimatum game task.

Figure 1 illustrates the time course of stimulus presentation in the present ultimatum game task, which was an adaptation of the task used in a previous study^4^. At the beginning of each trial, subjects were informed about the source of the offer, that is, either the computer, or a previous player (human). This information was presented on the screen for 1000 ms, followed by a central fixation cross presented for 900 to 1300 ms. Subsequently the offer screen was presented for 2000 ms, depicting a schematic illustration of the proposed distribution of points between the respective proposer and the subject. Next, participants were asked to accept or reject the offer by button press, with 3000 ms maximum response time. If subjects did not respond within this time window, they were asked to respond faster (speed-up screen presented for 1300 ms) and the trial was aborted. After a response had been logged, a fixation cross appeared for 800 to 1200 ms before feedback was presented for 2000 ms. Feedback screens were designed to visually match the offer screens as closely as possible while containing information about the exact amount of points awarded to each party. Trials were separated by an inter-trial interval of 500 ms.

In accordance with previous studies^4^,^12^, offers could be equitable/fair (50:50 or 40:60), inequitable/unfair (10:90 and 20:80), or moderately inequitable (30:70) toward the recipient. Unbeknownst to the subjects, all offers were actually generated by the computer and presented in randomized order. Frequency of the different offers and offer sources (computer/human) was balanced. As outlined above, participants completed two runs of the task in two separate sessions, and each run comprised a total of 300 trials organized in six blocks that were separated by short breaks. Runs took about 45 minutes each, including all breaks.

At the end of each run, subjects also completed a proposer version of the task that was specifically introduced to complement the cover story. This task took about 3 minutes and comprised 25 trials in which subjects were simply asked to make offers that would allegedly be fed into the pool of offers available for future test sessions. Participants were informed that offers in the proposer version did not count towards their final scores as it was unknown if they would be accepted by future subjects.

### Psychophysiological recordings

EEG was recorded only during the receiver version of the ultimatum game task. EEG was recorded from 64 electrodes using a BioSemi active electrode system (BioSemi B.V., Amsterdam, Netherlands) with a sampling rate of 512 Hz using the accompanying ActiView software package. Instead of ground and reference, the BioSemi EEG system uses a CMS/DRL feedback loop with two additional electrodes (for more information see: http://www.biosemi.com/faq/cms&drl.htm). Impedances were kept below 20 kΩ. Electrodes were mounted to an elastic cap according to the international 10–20 system. Vertical and horizontal eye movements were recorded with two electrodes attached above and beneath the left eye (VEOG) and two electrodes attached to the right and left outer canthi (HEOG).

EEG data were processed offline using BrainVision Analyzer 2 (Brain Product GmbH, Munich, Germany) and Matlab (Mathworks, Natick, Massachusetts, USA). Raw EEG data were filtered with a 30 Hz low-pass and 0.1 Hz high-pass filter. Ocular correction was performed using the Gratton & Coles^38^ algorithm as implemented in BrainVision Analyzer 2 using HEOG and VEOG channels. Automatic artifact rejection was performed based on the following criteria: maximum allowed voltage step 50 μV, maximal difference of values in intervals 150 μV, and minimal/maximal amplitudes of ± 200 μV. To obtain offer-locked ERPs, EEG data were segmented into 1200 ms epochs starting 200 ms before presentation of an offer, and baseline correction was performed based on the 200 ms preceding offer presentation. Segments were then averaged according to *condition* (observation/control), *source* (computer/human) and *fairness* (fair/unfair). Note that in accordance with prior work^4^ trials with moderately inequitable offers were not included in the analysis. For outcome-locked ERPs, only trials in which offers had been accepted entered analysis. EEG data were segmented into 4000 ms epochs starting with the button press reflecting the acceptance of an offer. Next, data were segmented into epochs from −200 to 1000 ms relative to feedback onset. Baseline correction was applied based on the 200 ms directly preceding presentation of the feedback screen. Segments were averaged according to *condition* (observation/control), *source* (computer/human) and *fairness* (fair/unfair). As for offer-locked ERPs, trials with moderately inequitable offers were not included.

As mentioned earlier, EEG data from one subject were excluded from analysis due to off-label use of a beta blocker, which has been shown to affect visually-evoked potentials^39^ and may hence affect stimulus-related ERPs. In one other subject, processed EEG data still contained excessive artefacts and were therefore excluded from ERP analyses. Furthermore, EEG data from subjects who had accepted fewer than five unfair offers from any source (human/computer) in any condition (observation/control) were also excluded, as the low number of single trial segments did not allow for meaningful averaging of feedback-locked segments. This affected five subjects, leaving a sample of N = 26 for ERP analyses.

After careful visual inspection of the stimulus-locked average and grand-average ERPs, FRN and P3 amplitudes for offer- and feedback-locked waveforms were defined as follows: FRN_offer_ amplitudes were measured as average amplitudes within the time window 280 to 330 ms after offer presentation at electrode FCz. Accordingly, the P3_offer_ was defined as average amplitude within the time window 400 to 600 ms after offer presentation at electrode Pz. FRN_feedback_ amplitudes were measured as average amplitudes within the time window 240 to 320 ms after feedback presentation at electrode FCz, and the P3_feedback_ was defined as average amplitude within the time window 400 to 600 ms after feedback presentation at electrode Pz.

### Analysis of behavioral and EEG data

Statistical analyses were performed using IBM SPSS 23 software (IBM Corporation, Armonk, New York, USA). Behavioral data (acceptance rates) as well as EEG data (FRN and P3 amplitudes for offer- and feedback locked ERPs) were analyzed by means of repeated measures analyses of co-variance (ANCOVAs) with *condition* (observation/control), *source* (computer/human), and *fairness* (fair/unfair) as within-subjects factors, and LSAS scores as covariate. Post-hoc paired-sample *t* tests were performed to resolve interactions when appropriate. If significant interaction effects involving the covariate emerged, difference scores were created for the measures of interest and correlated with LSAS scores. This is described in more detail in the respective paragraphs of the Results section.

## Results

### Behavior

On average, subjects scored 8170 ± 781 points (range 6370 to 9000 points) in the observation condition and 8332 ± 919 points (range 5150 to 9000 points) in the control condition. Table 1 shows individual scores in each condition for all subjects included in the behavioral analysis. Individual numbers of accepted offers according to fairness, condition, and source are provided in Table 2.

**Figure 2:**
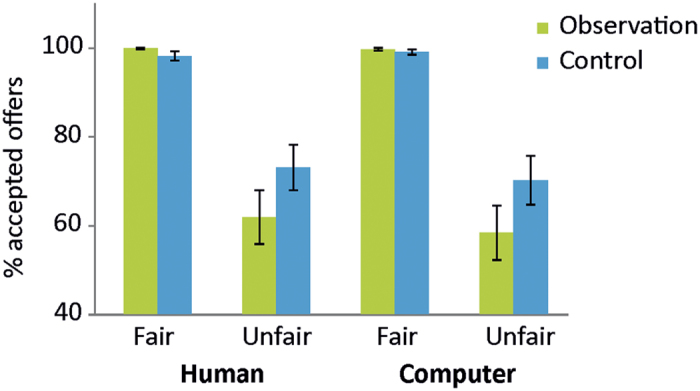
Mean acceptance rates according to fairness, source and condition.

Mean acceptance rates according to *fairness, source* and *condition* are provided in Fig. 2. The ANCOVA yielded a significant main effect of *fairness (F*_[1, 31]_ = 23.117, *p* < 0.0001), with higher acceptance rates for fair as compared to unfair offers. Furthermore, a significant *condition* by *fairness* interaction emerged (*F*_[1, 31]_ = 4.332, *p* = 0.046). Paired-sample *t* tests comparing acceptance rates between observation and control condition separately according to *fairness* revealed significantly lower acceptance rates under observation for unfair (t_32_ = −2.697, *p* = 0.011) but not for fair offers (*p* = 0.178). All other effects failed to reach significance (all *p* > 0.180).

### EEG

#### FRN_offer_

**Figure 3:**
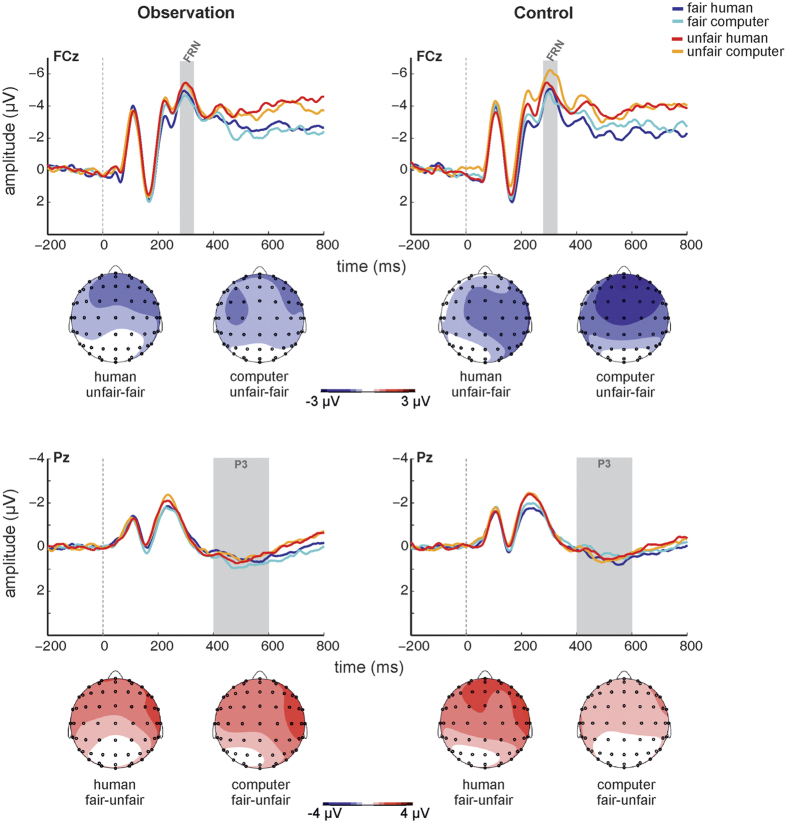
Offer-locked grand-average ERPs at electrodes FCz and Pz according to *source, fairness*, and *observation condition*, and scalp topographies of the unfair-fair difference signal in the FRN time window and the fair-unfair difference signal in the P3 time windows according to *source* and *observation condition*.

Figure 3 shows grand-average offer-locked ERPs at electrodes FCz and Pz according to *condition, source*, and *fairness*. Scalp topographies of the unfair-fair difference signal (Fig. 3) revealed typical fronto-central negativity in the FRN time window. The ANCOVA yielded a significant main effect of *fairness* (F_[1, 24]_ = 4.445, *p* = 0.046), with unfair feedback associated with more negative amplitudes, reflecting a larger FRN, relative to fair feedback (mean fair −4.64 μV ± 3.78; mean unfair −5.42 μV ± 3.94). No other significant main effects or interaction emerged (all *p* > 0.385).

#### P3_offer_

For P3_offer_ amplitudes, a trend but non-significant *condition* by *fairness* interaction emerged (F_[1, 24]_ = 3.373, *p* = 0.079). Scalp topographies of the fair-unfair difference signal (Fig. 3) showed broadly distributed positivity in the P3 time window. All other main effects and interactions were non-significant (all *p* > 0.189).

#### FRN_feedback_

**Figure 4:**
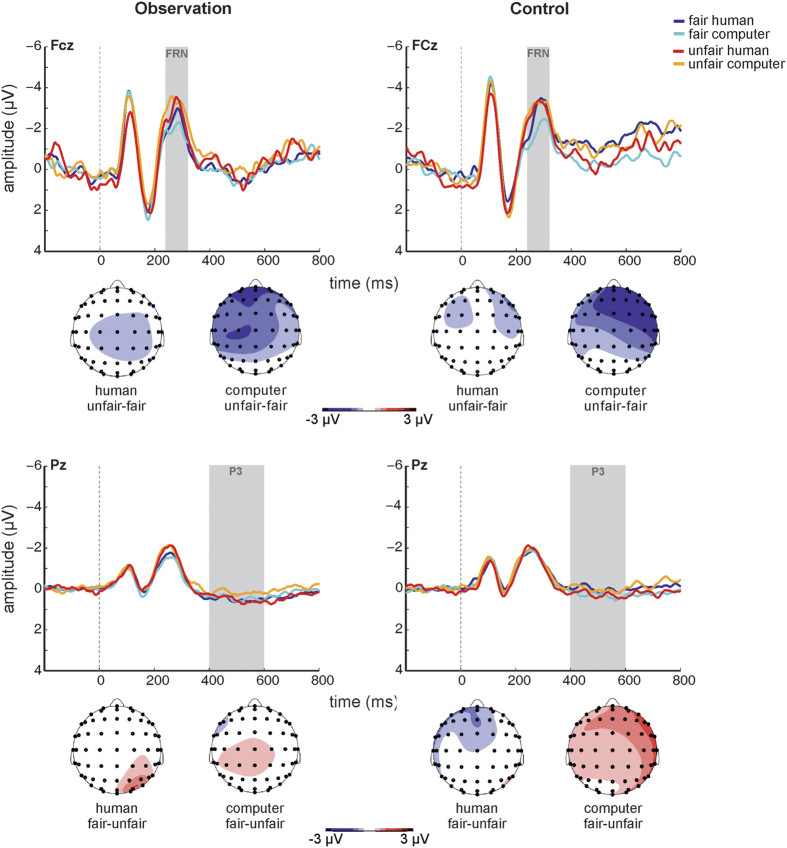
Feedback-locked grand-average ERPs at electrodes FCz and Pz according to *source, fairness*, and *observation condition*, and scalp topographies of the unfair-fair difference signal in the FRN time window and the fair-unfair difference signal in the and P3 time windows according to *source* and *observation condition*.

**Figure 5:**
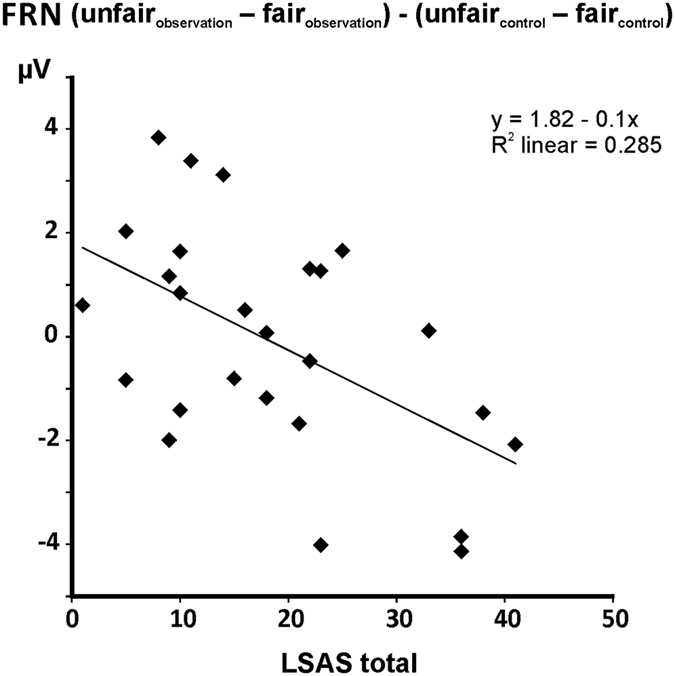
Scatter plots with regression lines illustrating the relationship between LSAS scores and FRN magnitude differences [(unfair_observation_ − fair_observation_) − (unfair_control_ − fair_control_)].

Figure 4 shows grand-average feedback-locked ERPs at electrodes FCz and Pz according to *condition, source*, and *fairness*. Scalp topographies of the unfair-fair difference signal (Fig. 4) revealed fronto-central negativity in the FRN time window that appeared more pronounced for computer offers. The ANCOVA yielded a significant main effect of *fairness* (F_[1, 24]_ = 6.631, *p* = 0.017), with unfair feedback associated with more negative amplitudes than fair feedback. The *condition* by *fairness* interaction was also significant (F_[1, 24]_ = 6.366, *p* = 0.019). Post-hoc paired-sample t tests comparing FRN amplitudes for fair and unfair feedback between conditions did not yield any significant differences (both *p* > 0.753). However, the alternative set of paired-sample t tests comparing FRN amplitudes between fair and unfair feedback separately in observation and control condition revealed significantly more negative amplitudes to unfair versus fair feedback in the observation condition (t_25_ = 3.314, *p* = 0.003; mean fair −2.13 μV ± 3.18; mean unfair −2.99 μV ± 3.03), and marginally also in the control condition (t_25_ = 2.008, *p* = 0.056; mean fair −2.29 μV ± 3.09; mean unfair −3.06 μV ± 2.51). Crucially, the *condition* by *fairness* interaction was further qualified by the covariate *LSAS score* (F_[1, 24]_ = 9.551, *p* = 0.005). In order to resolve this interaction, FRN magnitude differences according to *fairness* and *condition* were calculated as [(unfair_observation_ − fair_observation_) − (unfair_control_ − fair_control_)] and correlated with LSAS scores. Note that the resulting difference value was more negative when the unfair-fair difference was larger under observation. Scatter plots and linear regression lines are provided in Fig. 5. A significant negative correlation emerged (r = −0.534, *p* = 0.005), indicating that higher LSAS scores were associated with smaller (more negative) difference values which reflected stronger differential coding of fairness in the FRN under social observation. None of the other main effects or interactions reached significance (all *p* > 0.166).

#### P3_feedback_

Scalp topographies of the fair-unfair difference signal (Fig. 4) revealed at best weak positivity in the P3 time window. The ANCOVA did not yield any significant main effects or interactions for P3 amplitude (all *p* > 0.255).

## Discussion

The present study investigated audience effects on behavioral performance and processing of offers and feedback in the UG in relation to inter-individual differences in social anxiety. Results corroborated findings of negative reciprocity in the UG and showed that social observation increased rejection rates for unfair offers. Both offer- and feedback-locked FRN coded for fairness, with more negative amplitudes for unfair offers/feedbacks. Differential coding of fairness was augmented under social observation only in the feedback-locked FRN. P3 amplitudes following offers and feedback were not modulated by social observation or fairness, and alleged offer source (human/computer) did not affect behavior or neural responses. Interestingly, fairness and social observation modulated the feedback-locked FRN as a function of social anxiety level.

Higher rejection rates for unfair than for fair offers replicate previous findings^2^,^3^,^4^,^12^. It has been proposed that such negative reciprocity actually reflects prosocialilty in the subjects’ explicit decisions to lose out on potential gains for the sake of punishing unfair offers^2^. However, this notion cannot explain why rejection of unfair offers persists also in the absence of social interaction, that is, when nobody can be punished, as is the case for computer-proposed offers, albeit to a smaller extent than for human-proposed offers^4^,^10^,^11^,^12^. Moreover, it has been argued that negative reciprocity cannot be inseparably linked to positive reciprocity as the two sides of a fairness preference spectrum^40^. Indeed, participants’ tendencies to reject unfair offers in the UG do not correlate with their tendencies towards prosocial behavior in the Trust Game, the Dictator Game, or the Prisoner’s Dilemma, suggesting that results may be better explained in terms of avoiding the imposition of an inferior status^40^. This notion may also explain why fairness considerations are more prevalent in responders than proposers. If responders cannot tell that proposers were deliberately unfair, proposers tend toward greedier offers^3^,^8^,^9^.

Previous findings have highlighted social interaction as a factor influencing UG performance. Rejection rates for unfair offers were higher for human- as opposed to computer-proposed offers^4^,^12^. This effect could not be replicated in the present sample, possibly due to methodological differences. While subjects in previous studies had been informed that they would be playing the UG together with three other players allegedly seated in different rooms, subjects in the present study were informed that offers would be drawn from a pool of offers from previous subjects. These instructions thus emphasized that no other players were present at the time, possibly weakening the human-computer offer source distinction.

The most intriguing behavioral result was the impact of social observation on behavior. Previous studies could not explicitly assess the impact of social observation, as third parties were not included as designated observers but rather as inactive third players or potentially sanctioning third parties^3^,^19^,^20^. A priori, we had hypothesized two possible effects of social observation: stronger focus on task instructions which stressed maximization of own payoff, resulting in decreased rejection rates, or stronger emphasis on implicit social norms (fairness), resulting in increased negative reciprocity. In line with the latter, rejection rates for unfair offers increased under social observation. In view of the de-emphasized responder-proposer interaction in the present task design (see above), this result may suggest that implicitly the interaction component of the task was shifted away from the proposer to the observer who might have been perceived to judge the subjects’ character based on whether they behave according to implicit social norms.

Offer-locked ERPs showed fairness coding in the FRN_offer_, a finding that is consistent with previous work^4^,^21^,^22^,^26^. However, in contrast to these studies, the present data did not show modulation of the parietal P3_offer_ by fairness. Indeed, the parietal P3 appeared rather weak. Offer-locked grand-average ERPs suggested a more frontally distributed fairness effect in the typical P3 time window, which may correspond to the P3a component (for an integrative theory of P3a and P3b, see ref. 41). Alternatively, a more frontal distribution of positivity in the P3 time window may also be related to the fact that subjects were specifically instructed to withhold responses while the offer was still on the screen and to only respond when asked to. This may have essentially turned the offer screen into a nogo stimulus. Indeed, increased (frontal) P3 amplitudes have been shown for nogo as compared to go stimuli, likely due to increased evaluative demand during response inhibition related processing^42^,^43^.

Social observation did not affect offer-related ERP components, possibly because at that time in the task no overt response had occurred yet. Social observation may have shifted emphasis to the feedback, thereby augmenting attention to and processing of the FB stimulus. Last, as for behavior, offer source did not modulate neural responses, possibly because task instructions de-emphasized the distinction between computer and human as proposer.

Stronger attentional focus of feedback stimuli appears plausible in light of the observed fairness coding in the feedback-locked FRN. FRN_feedback_ amplitudes were less negative for fair relative to unfair offers. This is generally in line with increased FRN following unfavorable outcomes (e.g., refs 44,45) and confirms that the FRN may be considered as a neural marker of fairness processing^5^. Interestingly, fairness coding in the FRN_feedback_ was increased under social observation, possibly indicating that increased arousal sensitized the performance monitoring system. Importantly, the present data do not show evidence for differential effects of social observation on neural responses to favorable or unfavorable outcomes. This is particularly interesting in view of recent results suggesting that the observed “net” FRN is actually a product of two distinct processes, namely, a relative negativity in response to negative feedback, and a relative positivity in response to reward that is reduced or absent for unfavorable outcomes (e.g. refs 46, 47, 48, 49). Variations in FRN magnitude may depend more strongly on the reward-related response^50^,^51^. This notion was supported by a combined EEG-fMRI study in which single-trial measures of responses to positive (but not negative) feedback were coupled with brain activity in midfrontal and midcingulate cortex and ventral striatum^47^. Distinct processes underlying the “net” FRN may also map onto the distinction of stimulus-locked N2 and feedback-locked FRN as products of two distinct neural mechanisms, one that produces a negativity to infrequent events with variable scalp distribution and noradrenergic origin, and one that produces reward-related positivity and no-reward-related negativity with frontal–central scalp and dopaminergic origin^52^. While it appears conceivable that these two processes are differentially affected by social observation, this issue will need to be addressed in future studies since the present task was not designed for disentanglement of reward positivity and “non-reward negativity”.

Remarkably, in line with the hypotheses, FRN_feedback_ amplitudes were modulated by fairness and social observation as a function of social anxiety, with higher levels of social anxiety associated with stronger effects. This extends previous findings of increased (offer-locked) FRN to unfair versus fair offers in high but not low trait anxiety^4^. However, in this study, extreme groups were investigated. In contrast, subjects in the present sample represented a normal distribution of subclinical social anxiety. The strong correlation between social anxiety and neural fairness coding under observation in the present study strongly suggests that neural processing of fairness in the UG may be affected by social observation also in individuals with clinically relevant social anxiety. This notion should be addressed in future studies. Notably, while neural responses to fairness were augmented under observation as a function of social anxiety, this was not the case for behavior. This may suggests that while observation may have sensitized the performance monitoring system, this effect was not strong enough to also manifest in overt behavior. In view of evidence for altered behavioral UG performance in individuals with generalized anxiety disorder or panic disorder^53^, it stands to reason that behavioral effects may only emerge in populations with clinical levels of social anxiety or diagnosed social anxiety disorder.

Social observation, fairness and social anxiety were not linked to P3_feedback_ amplitude in the present study. In general, the P3 has been associated with decision making^23^ and stimulus evaluation^29^. As outlined above, previous studies have reported fairness effects in the offer-locked P3 (e.g., refs 4,26). It seems intuitive that evaluation and decision processing should be particularly important prior to response formation, and thus more sensitive to contextual modulation when related to the offer rather than the feedback stimulus.

## Conclusion

Taken together, the present study shows increased negative reciprocity and augmented fairness coding in the feedback-locked FRN in the UG under social observation. Notably, neural responses to fair versus unfair feedback were modulated by social observation as a function of social anxiety, with higher levels of social anxiety associated with augmented fairness coding. These results corroborate recent findings of altered ultimatum game performance and feedback processing in anxious individuals^4^,^12^,^51^. The present study is the first to show that a specifically targeted manipulation of social context (i.e., social observation) alters fairness processing in individuals with modest levels of social anxiety, lending support to the notion that that altered sensitivity to equality in a social context may contribute to social avoidance in socially anxious individuals^4^.

## Additional Information

**How to cite this article:** Peterburs, J. *et al*. Processing of fair and unfair offers in the ultimatum game under social observation. *Sci. Rep.* **7**, 44062; doi: 10.1038/srep44062 (2017).

**Publisher's note:** Springer Nature remains neutral with regard to jurisdictional claims in published maps and institutional affiliations.

## Figures and Tables

**Table 1:** Individual subjects’ scores in observation and control condition of the UG task.

Subject	Observation	Control
*1*	7180	6470
*2*	8840	9000
*3*	7570	8350
*4*	8680	8350
*5*	6700	8450
*6*	8940	8710
*7*	7960	8130
*8*	8960	8630
*9*	7460	8480
*10*	8440	9000
*11*	8910	8830
*12*	7280	8960
*13*	8620	8440
*14*	8990	8790
*15*	9000	8980
*16*	8500	8950
*17*	8870	9000
*18*	8550	8090
*19*	8880	8980
*20*	7820	8590
*21*	8930	9000
*22*	8610	6590
*23*	8520	8790
*24*	8590	8830
*25*	8230	9000
*26*	9000	9000
*27*	8390	9000
*28*	7990	8130
*29*	7430	5150
*30*	7200	7210
*31*	6370	8480
*32*	6690	6660
*33*	7510	7920

**Table 2:** Individual numbers of accepted trials in each condition.

Subject	Observation				Control			
	Human		Computer		Human		Computer	
	*fair*	*unfair*	*fair*	*unfair*	*fair*	*unfair*	*fair*	*unfair*
*1*	60	16	60	16	57	5	58	10
*2*	59	59	60	58	60	60	60	60
*3*	60	29	60	22	59	48	58	39
*4*	60	30	60	58	60	25	60	51
*5*	60	9	60	15	60	22	60	53
*6*	60	56	60	58	59	49	59	53
*7*	60	22	60	17	59	32	58	36
*8*	60	60	59	60	60	50	59	50
*9*	57	17	60	15	60	50	59	29
*10*	58	49	59	49	60	60	60	60
*11*	59	60	59	60	60	53	60	56
*12*	54	23	57	34	60	60	60	59
*13*	60	41	60	42	60	34	60	33
*14*	60	59	60	60	59	56	59	54
*15*	60	60	60	60	60	59	60	59
*16*	60	39	60	38	60	55	60	60
*17*	60	57	60	58	60	60	60	60
*18*	60	50	60	40	60	30	60	31
*19*	60	55	60	55	60	60	60	58
*20*	60	11	59	30	60	42	60	43
*21*	60	58	59	60	60	60	60	60
*22*	60	48	60	39	50	22	50	24
*23*	59	41	60	41	60	52	60	47
*24*	60	47	59	45	60	57	60	47
*25*	60	25	60	28	60	60	60	60
*26*	60	60	60	60	60	60	60	60
*27*	60	39	60	36	60	60	60	60
*28*	59	5	60	54	59	25	59	39
29*	60	7	60	5	57	0	42	0
30*	60	0	60	0	60	2	60	0
31*	60	3	58	0	60	35	60	42
32*	60	1	60	5	54	23	54	27
33*	60	15	60	2	60	18	60	21

*Subjects 29–33 were excluded from EEG analysis due to fewer than 5 accepted offers in at least one condition.
